# Metformin attenuates fibroblast activation during pulmonary fibrosis by targeting S100A4 *via* AMPK-STAT3 axis 

**DOI:** 10.3389/fphar.2023.1089812

**Published:** 2023-02-03

**Authors:** Huimin Ji, Hongliang Dong, Yuejiao Lan, Yuqian Bi, Xuan Gu, Yongyue Han, Chongyang Yang, Minghan Cheng, Jian Gao

**Affiliations:** ^1^ Pediatric Translational Medicine Institute, Shanghai Children’s Medical Center, School of Medicine, Shanghai Jiao Tong University, Shanghai, China; ^2^ The Second Affiliated Hospital, Dalian Medical University, Dalian, Liaoning, China; ^3^ Jilin Province People's Hospital, Changchun, Jilin, China; ^4^ 3201 Hospital, Hanzhong, Shaanxi, China

**Keywords:** pulmonary fibrosis, metformin, fibroblast activation, S100A4, STAT3

## Abstract

Fibroblasts activation is a crucial process for development of fibrosis during idiopathic pulmonary fibrosis pathogenesis, and transforming growth factor (TGF)-β1 plays a key regulatory role in fibroblast activation. It has been reported that metformin (MET) alleviated bleomycin (BLM)-induced pulmonary fibrosis (PF) by regulating TGF-β1-induced fibroblasts activation, but the underlying mechanisms still deserve further investigations. In this study, MET blocked α-smooth muscle actin (α-SMA) accumulation *in vivo* accompanied with S100A4 expression and STAT3 phosphorylation inhibition, resulting in attenuating the progression of lung fibrosis after BLM administration. We determined that S100A4 plays critical roles in fibroblasts activation *in vitro*, evidenced by siRNA knockdown of S100A4 expression downregulated TGF-β1 induced α-SMA production in Human fetal lung fibroblast (HFL1) cells. Importantly, we found for the first time that the expression of S100A4 in fibroblasts was regulated by STAT3. Stattic, an effective small molecule inhibitor of STAT3 phosphorylation, reduced S100A4 level in TGF-β1- treated HFL1 cells accompanied with less α-SMA production. We further found that MET, which inhibits STAT3 phosphorylation by AMPK activation, also inhibits fibroblasts activation by targeting S100A4 *in vitro*. Together all these results, we conclude that S100A4 contributes to TGF-β1- induced pro-fibrogenic function in fibroblasts activation, and MET was able to protect against TGF-β1-induced fibroblasts activation and BLM-induced PF by down-regulating S100A4 expression through AMPK-STAT3 axis. These results provide a useful clue for a clinical strategy to prevent PF.

## 1 Introduction

Pulmonary Fibrosis (PF) is a chronic progressive lung interstitial disease, characterized by distorted alveolar structure, excessive proliferation of lung mesenchymal cells and abnormal deposition of extracellular matrix (ECM), which results in reduced lung compliance, gas exchange impairment, and irreversible decline in pulmonary function (Yang et al., 2019; Kasam et al., 2020). PF with unknown etiology is termed idiopathic pulmonary fibrosis (IPF). The incidence of idiopathic pulmonary fibrosis (IPF) is related to gender (male: female ratio, 7:3) and age (Raghu et al., 2018; Wijsenbeek and Cottin, 2020). Nearly half of IPF patients die from respiratory failure on average within 2–4 years after diagnosis (Richeldi et al., 2017). The COVID-19 pandemic rapidly spreads around the world, and it ranges in severity from asymptomatic through to severe acute respiratory distress, which can lead to respiratory failure and death. It has rapidly become evident that COVID-19 patients infected with SARS-CoV-2 virus can develop features of interstitial pulmonary fibrosis (John, Joseph, Jenkins and Tatler, 2021). In recent years, pirfenidone and nintedanib, two drugs approved from Food and Drug Administration (FDA), are available for treating IPF (Somogyi, Chaudhuri, Torrisi, Kahn, Muller and Kreuter, 2019). However, neither of them provides a cure, and both of them are associated with several serious drug-related side effects, including gastrointestinal events, rash and photosensitivity (Richeldi et al., 2014; Cottin and Maher, 2015; Zhang et al., 2019). Therefore, it is an urgent need to elucidate the molecular mechanism and key targets of PF for developing effective therapeutic drugs. A large number of studies support the view that fibroblasts activation plays a crucial role in the progression of PF. During fibroblasts activation, lung-resident fibroblasts proliferate and differentiate into contractile mesenchymal cells, named myofibroblasts, which secret α-smooth muscle actin (α-SMA), and participate in excessive deposition of ECM and distortion of alveolar architecture (Kuhn and McDonald, 1991; Sontake et al., 2017; Wijsenbeek and Cottin, 2020).

S100A4, a calcium-binding protein, also termed fibroblast-specific protein-1(FSP-1), was previously considered as a marker of fibroblasts, regulating cellular biological functions, such as cell mobility, proliferation, or metastasis (Bresnick et al., 2015). In addition, S100A4 is involved in the pathogenesis of inflammation, autoimmune diseases and fibrosis (Fei, Qu, Li, Wang, Li and Zhang, 2017; Austermann et al., 2018). Increased expression levels of S100A4 have been reported in the lungs of IPF patients, including in mesenchymal progenitor cells, suggesting the involvement of S100A4 in IPF pathogenesis through modulating mesenchymal progenitor cell fibrogenicity (Xia et al., 2017; Lee et al., 2020). Like other members of the S100 family, S100A4 has intracellular as well as extracellular functions. Extracellular S100A4 produced and released by macrophages has been proved to be a key driver of lung fibroblast activation (Li et al., 2018; Zhang et al., 2018; Li et al., 2020), but the roles of intracellular S100A4 produced within fibroblasts in the process of fibroblast activation are still unclear.

Signal transducer and activator of transcription 3 (STAT3), a member of STATs family, is activated by multiple cytokines, including interleukin-6 and transforming growth factor (TGF)-β1 (Chakraborty et al., 2017). Upon binding of these ligands to their receptors, STAT3 is activated by phosphorylation at Tyr-705 in the STAT3 transactivation domain, and translocates to the nucleus, modulating the transcription of target genes (Bharadwaj, Kasembeli, Robinson and Tweardy, 2020). Activated STAT3 is elevated in the fibrotic lungs of patients with IPF and the Bleomycin (BLM) -induced mice PF model. On the other hand, STAT3 contributes to activating fibroblasts to transform into myofibroblasts, finally leading to abnormal accumulation of ECM (Pedroza et al., 2016). Therefore, STAT3 may be a potential therapeutic target in PF.

Many studies have reported the therapeutic effects of MET in PF. It has been suggested that intraperitoneal administration of MET attenuates BLM-induced lung fibrosis in mice *via* NADPH oxidase 4 (NOX4) suppression (Sato et al., 2016). MET reversed established lung fibrosis in both BLM- or silica-induced PF model, suggesting activation of AMP-activated protein kinase (AMPK) as key underlying signaling event, leading to downregulation of α-SMA and collagen (Rangarajan et al., 2018; Cheng et al., 2021). MET alters the fate of myofibroblasts and accelerates fibrosis resolution by inducing myofibroblast-to-lipofibroblast transdifferentiation (Kheirollahi et al., 2019). Our previous research showed that MET suppressed the proliferation of fibroblasts in PF by AMPK (Gu et al., 2021). Given that proliferation is a process of fibroblast activation, it is worth to further explore the effects of MET on fibroblasts activation in PF and its underlying molecular mechanisms. In this study, we established the BLM-induced PF model *in vivo* and TGF-β1-induced fibroblasts activation model *in vitro,* and found that MET was able to alleviate TGF-β1-induced fibroblasts activation and BLM-induced mice PF by down-regulating S100A4 expression through AMPK-STAT3 axis.

## 2 Materials and methods

### 2.1 Chemicals and materials

Bleomycin (Cat. No. 20026111) was purchased from Hanhui Pharmaceuticals Co., Ltd. Metformin (Cat. No. D150959) was purchased from Sigma-Aldrich. Stattic (Cat. No. HY-13818) was obtained from MedChemExpress, and Recombinant Human TGF-β1 (Cat. No. 100-21) was purchased from PeproTech.

### 2.2 BLM-induced PF model

This study performed on the mice conforms to the Guidelines from the National Institutes of Health and was approved by the Research Ethics Committee of Dalian medical University (animal ethics approval No. AEE19013). Male C57BL/6J mice (8–10 weeks of age, SPF grade) were purchased from Institute of Genome Engineered Animal Models for Human disease of Dalian Medical University. Mice were randomly divided into Saline groups, BLM groups, BLM + MET groups, and MET groups (*n* = 20 per group). Intratracheal instillation of BLM with 4.5 mg/kg (Gu et al., 2021) was performed to induce PF model in mice of BLM groups, and the mice of Saline groups received an equal volume of normal saline. Day 7 after administration of BLM or saline, mice in BLM + MET groups and MET groups were intraperitoneal injected with MET (65 mg/kg, Sigma, D150959) (Rangarajan et al., 2018) for every other day. At 21 days of BLM insult, mice lungs were collected for further assay.

### 2.3 Histology and immunohistochemistry

In brief, mice lungs were fixed in 4% paraformaldehyde and embedded in paraffin. Sequentially, lung was were sliced into 5 μm slices, then, lung slices were stain with Hematoxylin and Eosin (H&E) and Masson’s trichrome for observing the lung structure and collagen deposition. Lung slices were subjected to immunohistochemical (IHC) staining according to standard procedures. Briefly, lung slices were incubated with a primary antibody specific for S100A4 (Abcam, ab27957, 1:200) and p-STAT3 (Tyr705) antibody (Abcam, ab76315, 1:100) at 4°C overnight. Second day, lung slices were incubated with secondary antibodies and DAB solution. The Histology and protein level of lung slices were viewed under the microscope (Leica, DM 2000). The staining intensity was quantified using ImageJ software V.1.4.3.67.

### 2.4 Cell culture

Human fetal lung fibroblast (HFL1, Cat. No. SCSP5049) was purchased from the Chinese Academy of Sciences Cell Bank (Shanghai, China) and cultured in Ham’s F-12K (Procell, PM150910) medium supplemented with 10% fetal bovine serum (FBS) (Gibco, 10099141C) and 1% penicillin-streptomycin Solution (Hyclone, SV30010) in an incubator at 37°C with 5% CO_2_ atmosphere.

### 2.5 Transfection with small interfering RNA

When confluence of fibroblasts reached 60%–70% in the 6-well plates, S100A4 siRNA or scramble siRNA (negative control siRNA) mixed with Lipofectamine 2000 (Invitrogen, 11668-027) according to the manufacturer’s instructions. HFL1 cells were transfected with S100A4 siRNA or Scrambled siRNA separately before TGF-β1 stimulation. S100A4 siRNA and scramble siRNA were synthesized by GenePharma. The sequences of S100A4 siRNA were shown as follows: the forward primer was 5′-GCA​UCG​CCA​UGA​UGU​GUA​ATT-3′, and the reverse primer was 5′-UUA​CAG​AUC​AUG​GCG​AUG​CTT-3’.

### 2.6 Western blot analysis

Lung tissues or fibroblasts were lysed with RIPA lysis buffer (Beyotime, P0013B) containing Phenylmethanesulfonyl fluoride (PMSF) (Beyotime, ST506) and phosphatase inhibitor (Beyotime, P1081), then protein concentrations were measured using BCA protein assay kit (Beyotime, P0010S). Protein extracts mixed with loading buffer were separated by sulfate-polyacrylamide gel electrophoresis (SDS-PAGE, 10%–15%) and then were transferred to polyvinylidene difluoride (PVDF, 0.22 μm and 0.45 μm) membranes (Millipore). After being blocked with 5% skim milk, the membranes were incubated overnight at 4°C with primary antibodies, including anti-p-STAT3 (Abcam, ab27957, 1:1,000), anti-p-STAT3 (Tyr705) (Abcam, ab76315, 1:1,000), anti-STAT3 (Cell signaling technology, 30835S, 1:1,000), anti-AMPK (Cell signaling technology, 2,532, 1:1,000), anti-p-AMPK (Cell signaling technology, 2,535, 1:500), anti-SMA (Abcam, 5,694, 1:1,000), anti-Col-Ⅰ (Bioss, bs-10423R, 1:1,000) and anti-GAPGH (Cell signaling technology, 2,118, 1:1,500). On the second day, after being incubated with anti-rabbit IgG (H + L) (Invitrogen, 35568), the protein bands on the membranes were detected by the Oddessy Clx. GAPDH was used for normalizing the expressed level of relative protein.

### 2.7 Scratch wound healing assay

The cells were seeded in 6-well plates and grown to confluence. A linear wound was performed to each confluent monolayer using a pipette tip and washed 3 times with PBS. Thereafter, the cells were cultured with serum free medium (Gibco, 31985-070), and the images of scratches were captured at 0 h and 24 h. The area of the cell gap was determined by the ImageJ software V.1.4.3.67. The following equation was used to evaluate the migrated area (%): [(cell gap area at 0 h − cell gap area at 24 h)/cell gap at 0 h] × 100%.

### 2.8 Immunofluorescence analysis

Cells were fixed in 4% paraformaldehyde, then permeabilized with 0.3% Triton X-100 (Byotime, ST795), and blocked with goat serum (Dalian Meilunbio, MB4508). Thereafter, cells were incubated with primary antibodies for S100A4 (1:200), p-STAT3 (Tyr705, 1:500) or α- SMA (Santa Cruz, sc-53142, 1:50) respectively, overnight at 4°C and then combined with TRITC- conjugated goat anti-mouse IgG (ZSGB-BIO, ZF-0313, 1:100) or FITC-conjugated goat anti-rabbit IgG (ZSGB-BIO, ZF-0311; 1:100). Cell nuclei were stained with DAPI (Byotime, C1005, 1:100) for 5 min at room temperature. The fluorescence images were captured by Inverted fluorescence Microscope (Leica, DMI3000B).

### 2.9 Statistical analysis

Data are present as mean ± standard deviation (SD) for three independent experiments. Data of two groups are analyzed using Student test (*t*-test), and data of multiple groups are analyzed by one-way analysis of variance (ANOVA). All statistical analysis is performed by SPSS software and column graphs are using GraphPad prism 8.3. In all cases, *p* < 0.05 was considered statistically significant.

## 3 Results

### 3.1 MET mediated anti-fibrosis effects in BLM-induced PF model accompanied with downregulation of S100A4 and phosphorylation of STAT3 expression

**FIGURE 1:**
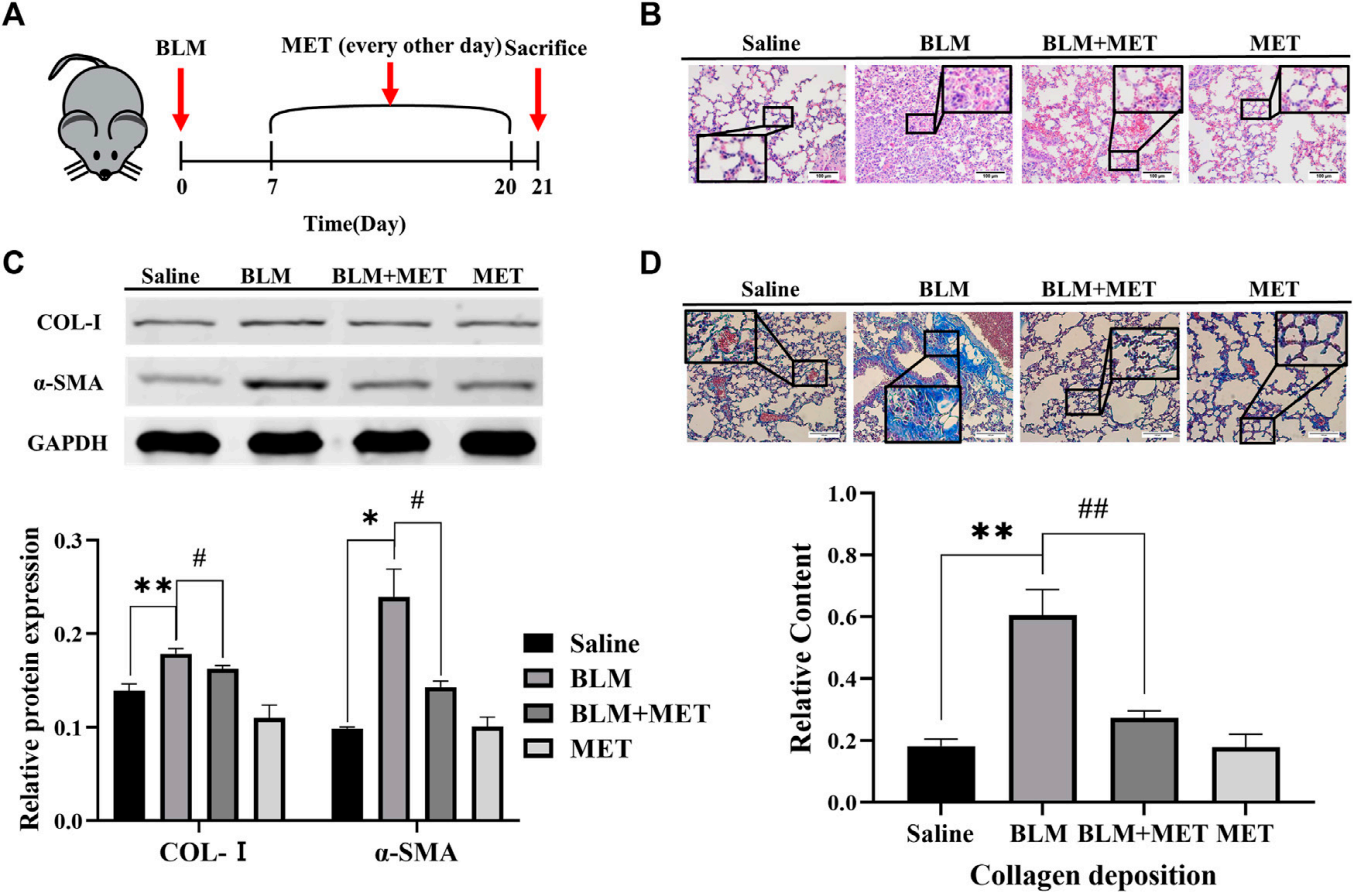
MET protected against Pulmonary fibrosis induced by BLM in C57BL/6J mice. **(A)** C57BL/6J mice were intraperitoneally given either MET (65 mg/kg) or normal saline every other day after administrated saline or BLM (4.5 mg/kg, *n* = 3). **(B)** Lung tissues of mice were collected after 21 days of intratracheal instillation of BLM or normal saline. Lung histology was observed through H&E staining. Scale bars represent 100 μm. **(C)** The expression level of Col-I and α-SMA from mice lung was measured by Western Blot analysis. **(D)** Masson’s trichrome staining was for observing collagen deposition. Scale bars represent 100 μm. Data are shown as the mean ± SD (*n* = 3, per group). ^*^ *p* < 0.05, ^**^ *p* < 0.01, compared with the saline group. ^*#*^ *p* < 0.05, ^*##*^ *p* < 0.01 compared with the BLM group.

MET has been known to mediate anti-fibrosis effects in PF, but its underlying mechanisms deserves further investigation (Sato et al., 2016; Rangarajan et al., 2018; Cheng et al., 2021). In this study, we first confirmed MET-mediated anti-fibrosis effects *in vivo* by employing BLM-induced PF model (Figure 1A). HE staining results showed that MET significantly ameliorated BLM induced severe disruption of alveolar structure, thicker alveolar septa, infiltration of inflammatory cells (Figure 1B). The expression of collagen I (Col-Ⅰ) and α-SMA in lung tissues, which were hallmarks of the degree of PF, were inhibited by MET treatment (Figure 1C). Masson staining results showed that MET treatment rescued BLM-induced collapse of alveolar spaces and pulmonary interstitial collagen deposition (Figure 1D). Together these data indicated that MET alleviated BLM-induced PF in C57BL/6J mice, which is consistent with previous findings (Sato et al., 2016; Rangarajan et al., 2018).

**FIGURE 2:**
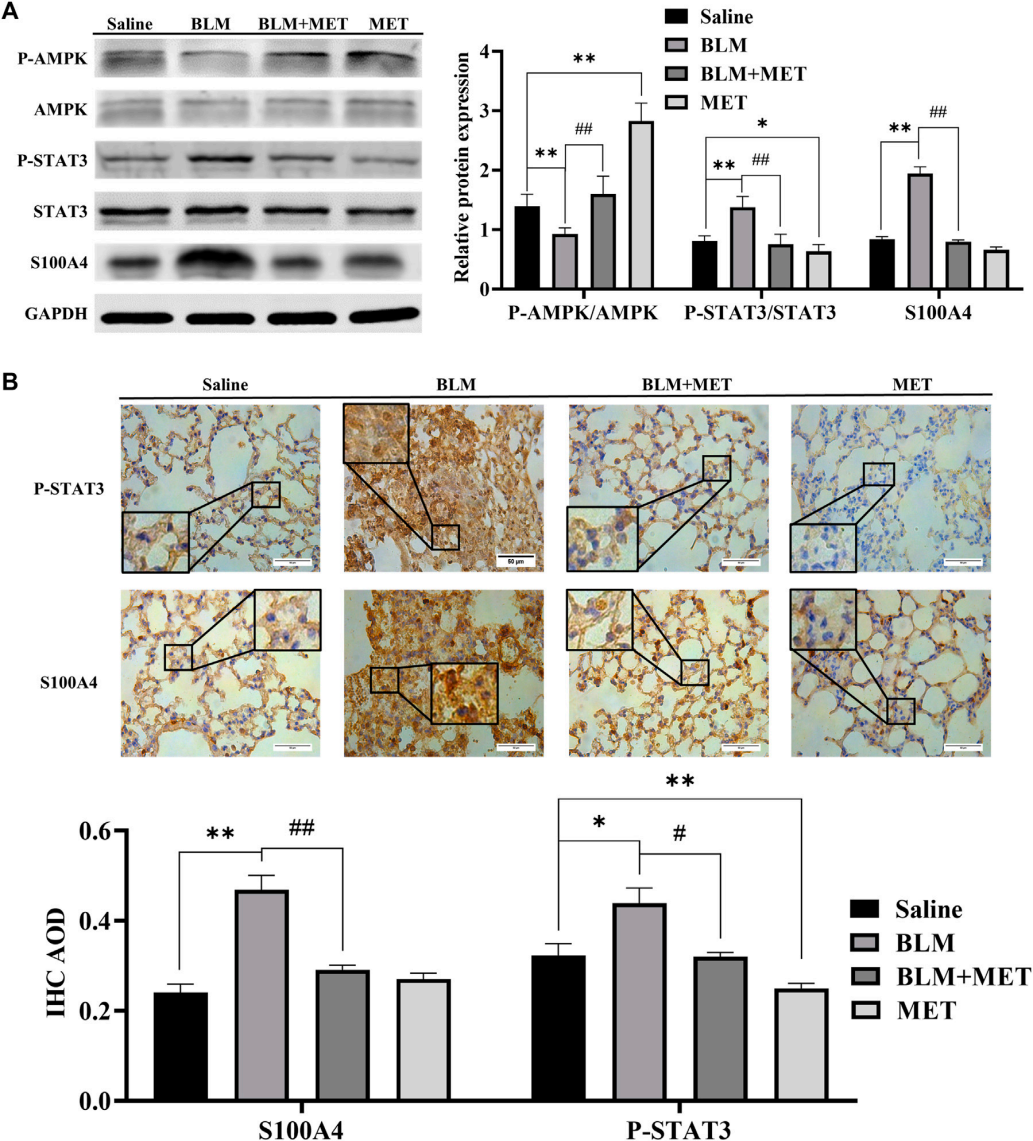
MET downregulated the expression of S100A4 and STAT3 Activation in BLM-induced PF mice. **(A)** Western blot analysis in lung using AMPK, p-AMPK, STAT3, p-STAT3 and S100A4 antibodies. **(B)** Representative images of IHC-stained lung section from mice using S100A4 and p-STAT3 antibodies, respectively. Scale bars represent 50 μm. Data were shown as mean ± SD (*n* = 3 per group). ^*^ *p* < 0.05, ^**^ *p* < 0.01, compared with the Saline group; ^*##*^ *p* < 0.01, compared with the BLM group.

Based upon the key roles of S100A4 in PF process (Xia et al., 2017; Lee et al., 2020), we further investigated the regulatory effects of MET on the expression of S100A4 in lung tissues of BLM-induced PF mice. Both the WB (Figure 2A) and IHC (Figure 2B) data showed that the protein level of S100A4 was significantly increased after BLM treatment, but MET administration reversed this change, indicating that MET mediated anti-fibrosis in PF by targeting S100A4. Notably, in lung tissues of BLM-induced PF model, BLM treatment induced STAT3 phosphorylation level upregulation and AMPK signaling inhibition also reversed by MET administration (Figures 2A, B). It is reasonable to speculate AMPK pathway, and STAT3 phosphorylation contributes to MET-mediated anti-fibrosis effects, but whether MET downregulated the expression of S100A4 *via* activating AMPK pathway and STAT3 phosphorylation needs further investigations.

### 3.2 Knockdown of S100A4 attenuated TGF-β1-induced fibroblasts activation

**FIGURE 3:**
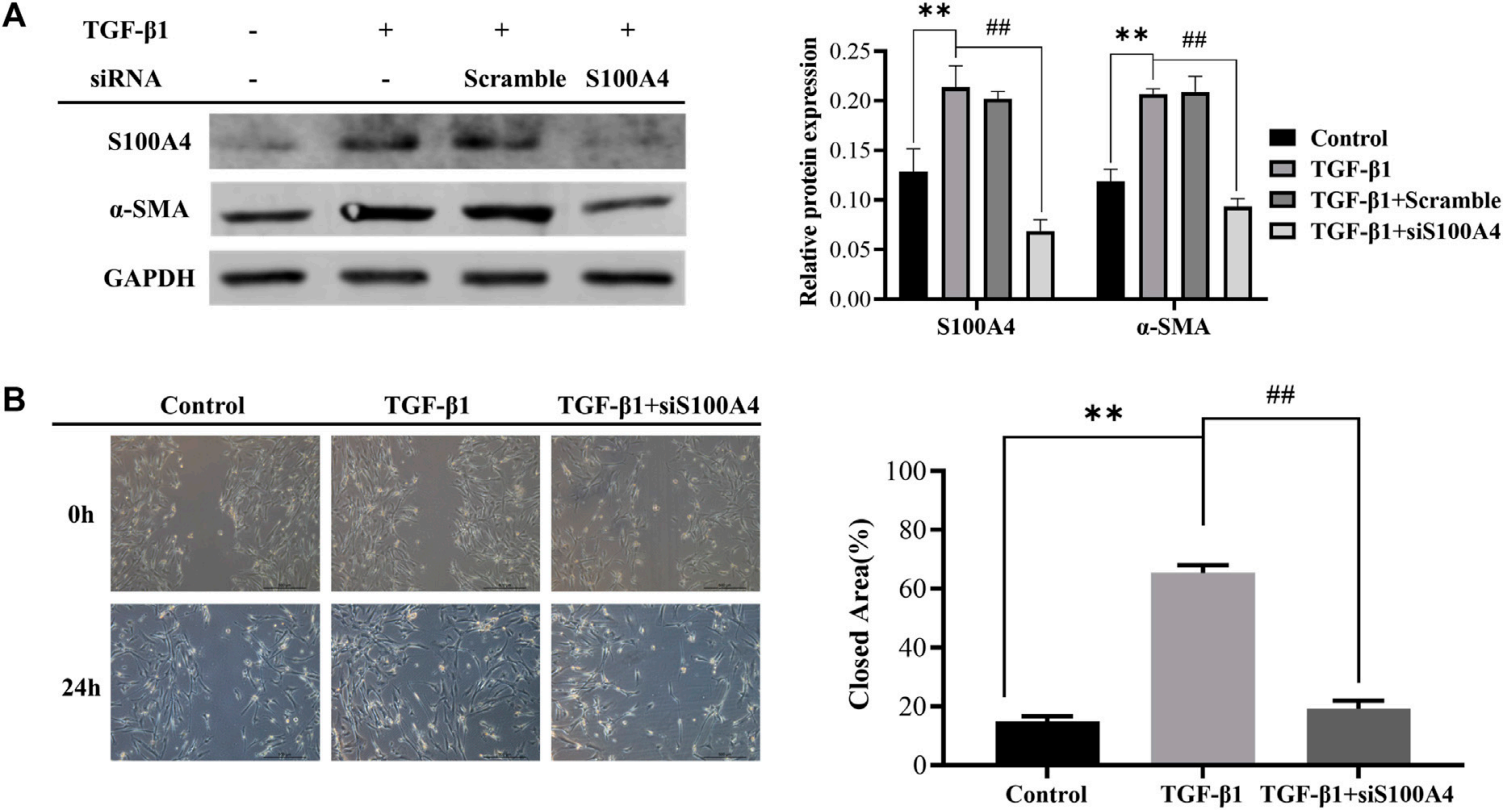
Knockdown of S100A4 attenuated TGF-β1-induced fibroblasts activation. HFL1s were transfected by S100A4 siRNA or Scrambled siRNA, and then stimulated with TGF-β1 (10 ng/mL) for 24 h. **(A)** The protein expression levels of α-SMA and S100A4 were measured by western blot in fibroblasts. **(B)** The migration abilities of fibroblasts were detected by scratch assay at 0 h and 24 h after treatment with TGF-β1, respectively. The closed area (%) of the scratch was calculated by equation: [(cell gap area at 0 h - cell gap area after 24 h)/cell gap area at 0 h] ×100%. Scale bars represent 500 μm. Data were shown as mean ± SD (*n* = 3), ^**^ *p* < 0.01 compared with Control group; ^*##*^ *p* < 0.01 compared with TGF-β1 group.

Differentiation of fibroblasts and production of collagen caused by TGF-β1 contribute to the pathogenesis of PF (Wynn and Ramalingam, 2012). In this work, we treated lung fetal fibroblasts (HFL1) with TGF-β1 (10 ng/mL) for 24 h to induce fibroblast activation *in vitro*. We found that TGF-β1 increased the expression of S100A4 (Figure 3A), which is consistent with the changes of α-SMA expression, a hallmark of differentiation of fibroblasts into myofibroblasts. These data indicated that S100A4 expression is positively related to fibroblasts activation and actively involved in PF. In order to further confirm the roles of intracellular S100A4 in fibroblasts activation, we knockdown S100A4 expression in TGF-β1 treated HFL1 cells using small interfering RNA, and the results showed that knockdown the expression of S100A4 reduced TGF-β1 induced α-SMA upregulation (Figure 3A) and inhibited migration abilities of fibroblasts induced by TGF-β1 (Figure 3B). Collectively, these results indicated that S100A4 contributes to TGF-β1 induced α-SMA accumulation in fibroblasts activation, which plays critical roles in PF pathology. Targeting S100A4 might provide us a potential therapeutic strategy for PF.

### 3.3 Inhibition of STAT3 activation alleviated TGF-β1 mediated S100A4 expression and fibroblast activation

**FIGURE 4:**
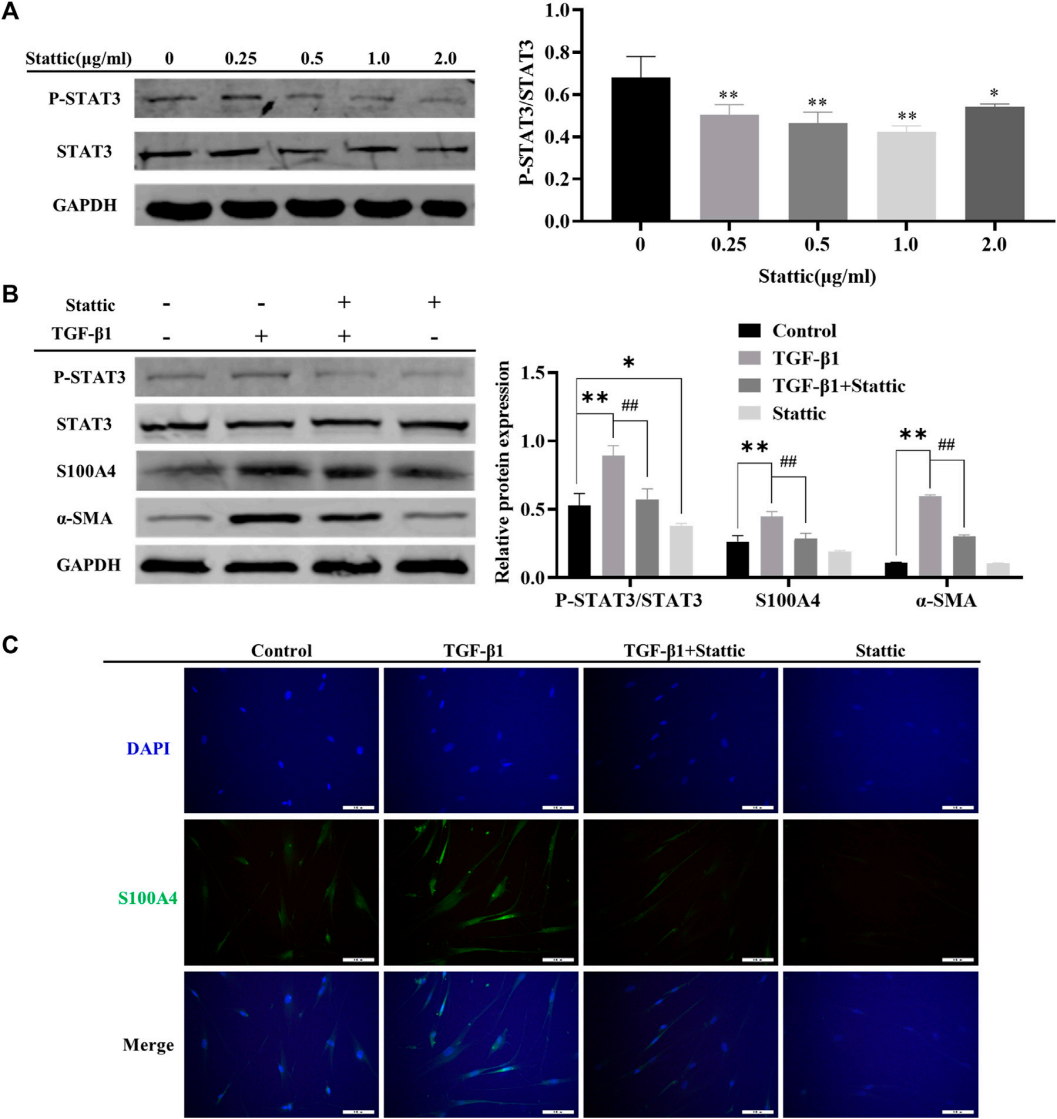
Inhibition of STAT3 activation alleviated TGF-β1 mediated S100A4 expression and fibroblast activation. **(A)** HFL1 cells were treated with different concentrations of Stattic (0 μg/mL–2.0 μg/mL) for 48 h. The phosphorylation level of STAT3 was measured by Western Blot. **(B, C)** Stattic treatment was started 24 h before TGF-β1 stimulation and the expression of protein were measured by western blot and immunofluorescence after 24 h treatment with TGF-β1. Scale bars represent 100 μm. Data were shown as mean ± SD (*n* = 3), ^*^ *p* < 0.05, ^**^ *p* < 0.01, compared with Control group; ##*p* < 0.01, compared with TGF-β1 group.

As shown in Figures 2A, B, MET downregulated the expression of S100A4 accompanied with AMPK pathway activation and STAT3 phosphorylation (Figures 2A, B). It has been reported that MET inhibits phosphorylation of STAT3 by activating AMPK(Lee et al., 2017; Ge, Wang, Miao and Yan, 2018; Bharadwaj, Kasembeli, Robinson and Tweardy, 2020). However, whether S100A4 expression in fibroblasts is regulated by phosphorylation of STAT3 is still unknown. As shown in Figure 4A, static inhibited STAT3 phosphorylation effectively and downregulated TGF-β1 induced the expression of S100A4 and fibroblasts activation (using α-SMA as a hallmark) in HFL1 cells (Figures 4B, C). All of these results indicated that inhibition of STAT3 activation alleviates fibroblast activation by regulating S100A4.

### 3.4 MET suppressed fibroblasts activation by targeting S100A4 *via* AMPK-STAT3 axis

**FIGURE 5:**
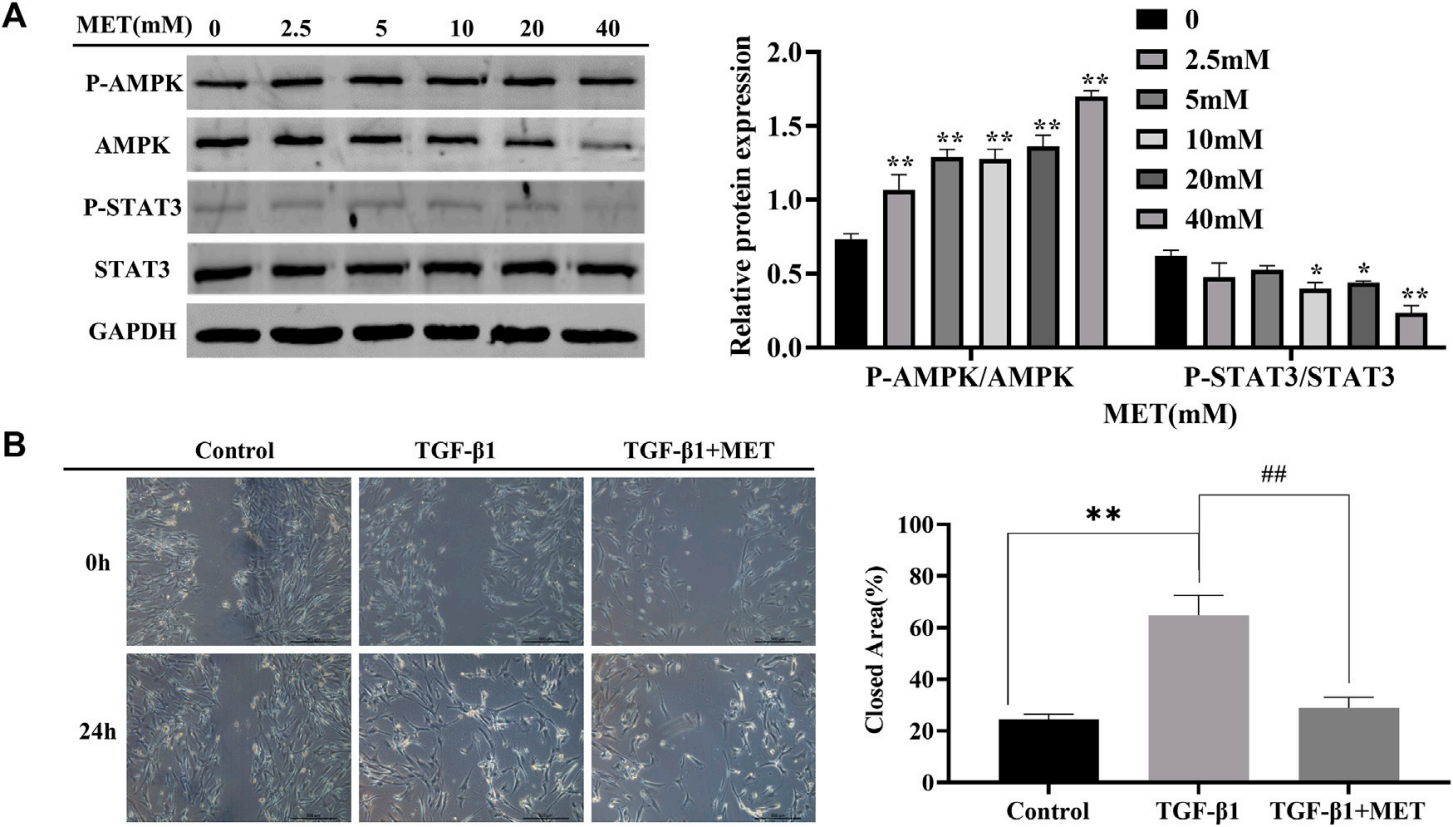
MET suppressed TGF-β1-induced fibroblasts activation. **(A)** HFL1 cells were treated with MET (0 mM–40 mM) for 24 h. Western blot was performed to measure the expression level of p-AMPK, AMPK, STAT3 and p-STAT3. **(B)** HFL1 cells were pretreated with MET (10 mM) for 24 h and then incubated with TGF-β1 (10 ng/mL) for 24 h. The migration abilities of fibroblasts were detected by scratch assay at 0 h and 24 h after treatment with TGF-β1, respectively. Scale bars represent 500 μm. The closed area (%) of the scratch was calculated by equation: [(cell gap area at 0 h - cell gap after 24 h)/cell gap area at 0 h] ×100%. **(C)** The expression of p-STAT3 and α-SMA were measured by immunofluorescence after 24 h treatment with TGF-β1. Scale bars represent 100 μm. Data were shown as mean ± SD (*n* = 3), ^*^ *p* < 0.05, ^**^ *p* < 0.01, compared with Control group; ^*##*^ *p* < 0.01, compared with TGF-β1 group.

Next, we wonder if MET-mediated downregulation of S100A4 expression is dependent on AMPK-STAT3 pathway. Firstly, AMPK activation and significant decrease of phosphorylated STAT3 in a dose dependent manner were observed after MET administration in HFL1 cells (Figure 5A). Furthermore, we found that increased migration abilities of fibroblasts induced by TGF-β1 stimulation were reversed by MET pretreatment (Figure 5B). Immunofluorescence data also further confirmed that MET pretreatment down-regulated α-SMA and STAT3 phosphorylation induced by TGF-β1 (Supplementary Figure S1). These data implied that MET exerts obvious inhibitory effects on fibroblasts activation by possibly regulating STAT3 phosphorylation.

**FIGURE 6:**
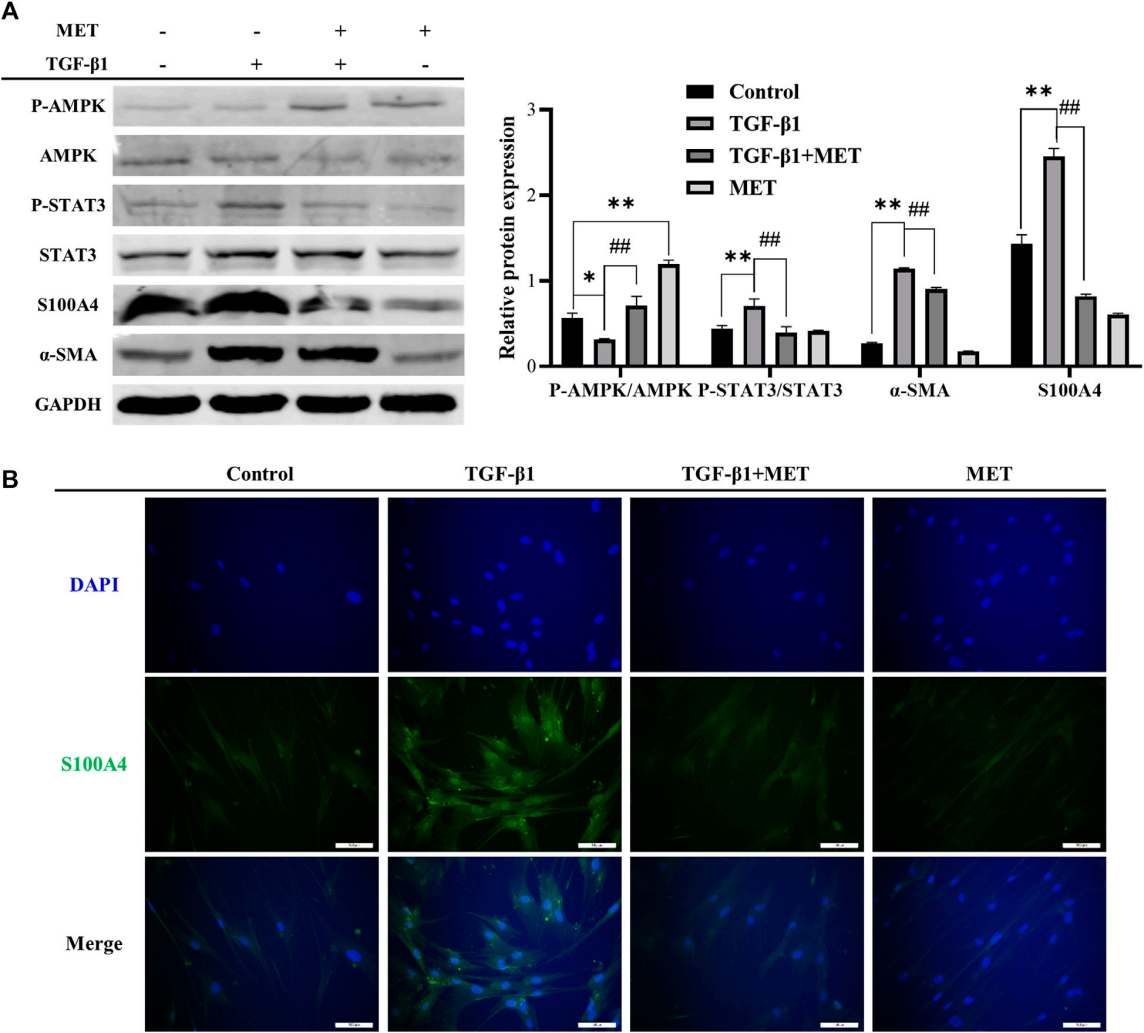
MET inhibited S100A4 *via* AMPK-STAT3 axis during fibroblasts activation *in vitro*. MET treatment was started 24 h before TGF-β1 stimulation. The expressions of protein were measured by western blot **(A)** and Immunofluorescence analysis **(B)**. Scale bars represent 100 μm. Data were shown as mean ± SD (*n* = 3), ^*^ *p* < 0.05, ^**^ *p* < 0.01, compared with Control group; ^*##*^<0.01, compared with TGF-β1 group.

In TGF-β1-induced fibroblasts activation system *in vitro*, the expression level of α-SMA, phosphorylated STAT3 and S100A4 in HFL1 cells increased. However, pretreatment with MET upregulated the protein level of activated AMPK, but significantly downregulated α-SMA, phosphorylated STAT3 and S100A4 (Figure 6A). Meanwhile, pretreatment of MET weakened the immunofluorescence activity of S100A4 induced by TGF-β1 (Figure 6B). Together all these data, we conclude that MET exerts inhibitory effect on fibroblast activation by targeting S100A4 *via* AMPK-STAT3 axis.

## 4 Discussion

IPF is a progressive interstitial lung disease characterized by fibroblasts activation and excessive ECM deposition. At present, only Nintedanib and Pirfenidone have been approved for treating IPF in Europe and the United States. However, serious adverse drug reaction limits their clinical applications (Richeldi et al., 2014; Cottin and Maher, 2015). It is necessary to explore the pathogenesis of IPF and develop novel therapeutic strategies. An important finding of this study is that fibroblasts activation can be inhibited by targeting S100A4 through downregulating STAT3 phosphorylation (Figures 4B, C), providing us a novel potential therapeutic strategies of IPF.

S100A4 was first known as specific marker of fibroblasts, and a great deal of evidence emphasizes the role of S100A4 in tissue fibrosis (Schneider et al., 2008; Louka and Ramzy, 2016; Qian et al., 2018). In fibrotic cardiac tissue, S100A4 is mainly expressed by hematopoietic cells and endothelial cells (Kong, Christia, Saxena, Su and Frangogiannis, 2013), while S100A4 secreted by subpopulation of macrophage activates hepatic stellate cells during liver fibrosis (Osterreicher et al., 2011; Chen et al., 2015). In PF, subpopulations of macrophages and fibroblasts are the major sources of S100A4 (Lawson et al., 2005; Li et al., 2018). Endonuclear S100A4 confers mesenchymal progenitor cell fibrogenicity through promoting p53 degradation in the progression of IPF (Xia et al., 2017). Previous studies mainly focus on extracellular S100A4 derived from macrophage promotes fibroblasts activation during PF (Li et al., 2018; Zhang et al., 2018; Li et al., 2020), while the regulatory effect of intracellular S100A4 on fibroblasts activation is still unclear. In the present study, we found S100A4 increased in lung tissues of BLM-induced PF mice and TGF-β1-treated HEF1 cells (Figures 2A, B), blocking S100A4 expression by siRNA exhibited anti-fibrosis effects (Figures 3A, B) or inhibiting STAT3 phosphorylation with a small molecule inhibitor static (Figures 4B, C), as well as MET (Figures 5, 6).

Therapeutic strategies by targeting S100A4 go beyond the field of lung fibrosis. Like in PF, S100A4, as a downstream mediator of the stimulatory effects of TGF-β1, amplifies TGF-β1-induced fibroblasts activation in systemic sclerosis (Tomcik et al., 2015). Silencing S100A4 with siRNA or blocking S100A4 with niclosamide inhibited fibroblasts activation in amyotrophic lateral sclerosis (Milani et al., 2021). S100A4 released from highly bone-metastatic breast cancer cells plays a critical role in osteolysis (Kim et al., 2019). Targeting S100A4 by employing chemotherapeutic drugs such as MET, antibodies, or small-molecule inhibitors might provide a new potential therapy for systemic sclerosi, amyotrophic lateral sclerosis and cancers.

As a member of STATs family, STAT3 is activated by multiple cytokines including interleukin-6 and TGF-β1 (Chakraborty et al., 2017). The level of STAT3 phosphorylation is elevated in the fibrotic lungs of patients with IPF and BLM-induced PF mice. On the other hand, STAT3 contributes to activating fibroblasts to transform into myofibroblasts, finally leading to abnormal accumulation of ECM (Pedroza et al., 2016). Previous studies show the role of STAT3 in tissue fibrosis, which activation of STAT3 contributes to PF and TGF-β1-induced fibroblast activation (Knight et al., 2011; Zehender et al., 2018). Stattic is a small non-peptide molecule that selectively targets the function of the tyrosinylation site (Tyr705) and the SH2 domain of STAT3, thereby inhibiting STAT3 phosphorylation and dimerization of activated STAT3 and nuclear transport (Schust, Sperl, Hollis, Mayer and Berg, 2006). Here, we reported that Stattic inhibited STAT3 activation and attenuated the fibroblasts activation by inhibiting S100A4 expression. Interestingly, macrophage-derived S100A4 activates STAT3 in hepatocytes followed by the upregulation of inflammatory factor gene expression, leading to inflammation, but suppresses lipid accumulation during chronic ethanol-induced fatty liver (Yuan et al., 2019). In PF, further study deserves to be carried out for investigating whether extracellular S100A4 activates STAT3 in fibroblasts, leading to fibroblasts activation.

Although many studies showed that the first-line antidiabetic drug, MET, is a potential therapeutic drug for PF (Rangarajan et al., 2018; Kheirollahi et al., 2019; Cheng et al., 2021), the underlying mechanisms needs to be further clarified. In this manuscript, we demonstrate that MET alleviated deposition of collagen, distorted alveolar structure in BLM-induced mice PF *in vivo*, and inhibited lung fibroblasts activation *in vitro* by targeting S100A4 *via* AMPK-ATAT3 axis. In addition, there were no abnormal findings in the vital signs and physical examination of the mice, suggesting to some extent that MET did not cause obvious adverse drug reaction in this study. Our findings provide novel important explanation for MET mediated a variety of favorable biological and therapeutic activities such as anti-fibrosis, anti-tumor, and anti-inflammation.

## 5 Conclusion

**FIGURE 7:**
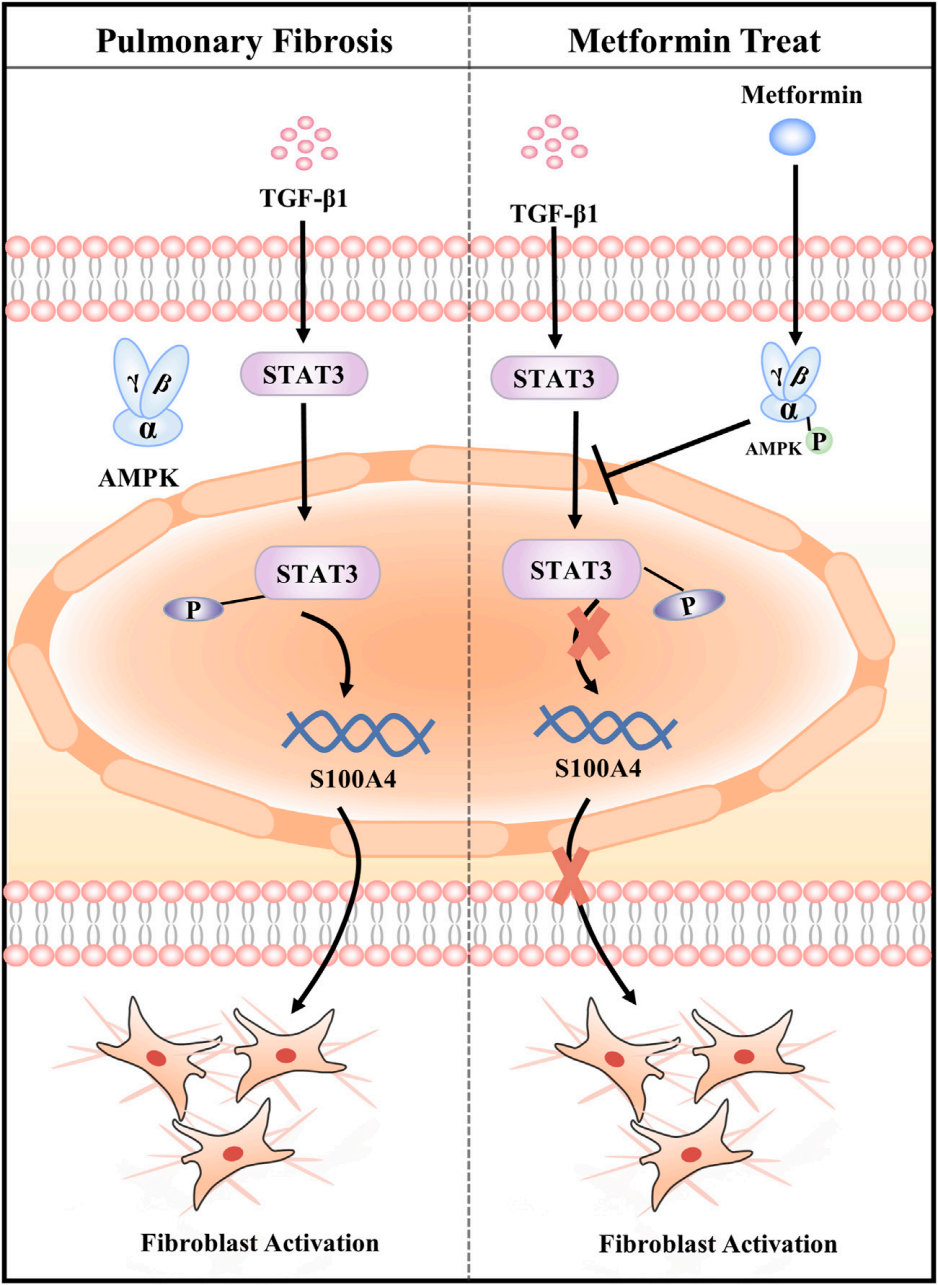
Graphical abstract of the mechanisms. Metformin attenuates Fibroblast Activation during Pulmonary Fibrosis by Targeting S100A4 *via* AMPK-STAT3 axis.

In summary, our data demonstrates that MET protects against BLM-induced PF of mice *in vivo* and attenuates TGF-β1-induced fibroblasts activation *in vitro* by targeting S100A4 *via* AMPK-STAT3 axis (Figure 7). Our study might provide a new potential therapy for S100A4-involved diseases including PF.

## Data Availability

The original contributions presented in the study are included in the article/Supplementary Material, further inquiries can be directed to the corresponding authors.

## Supplementary material

Supplementary Figure 1 HFL1 cells were pretreated with MET (10 mM) for 24 h and then incubated with TGF-β1 (10 ng/ml) for 24 h. The expression of P-STAT3 and α-SMA were measured by immunofluorescence after 24 h treatment with TGF-β1. Scale bars represent 100 μm.
