# Supplementary material for: Metformin attenuates fibroblast activation during pulmonary fibrosis by targeting S100A4 via AMPK-STAT3 axis
Source: Front Pharmacol. 2023 Feb 3;14:1089812. doi: 10.3389/fphar.2023.1089812 (PMC9936158; doi:10.3389/fphar.2023.1089812)
Supplement: Supplementary file 1 [file Image1.pdf]

Supplementary Material

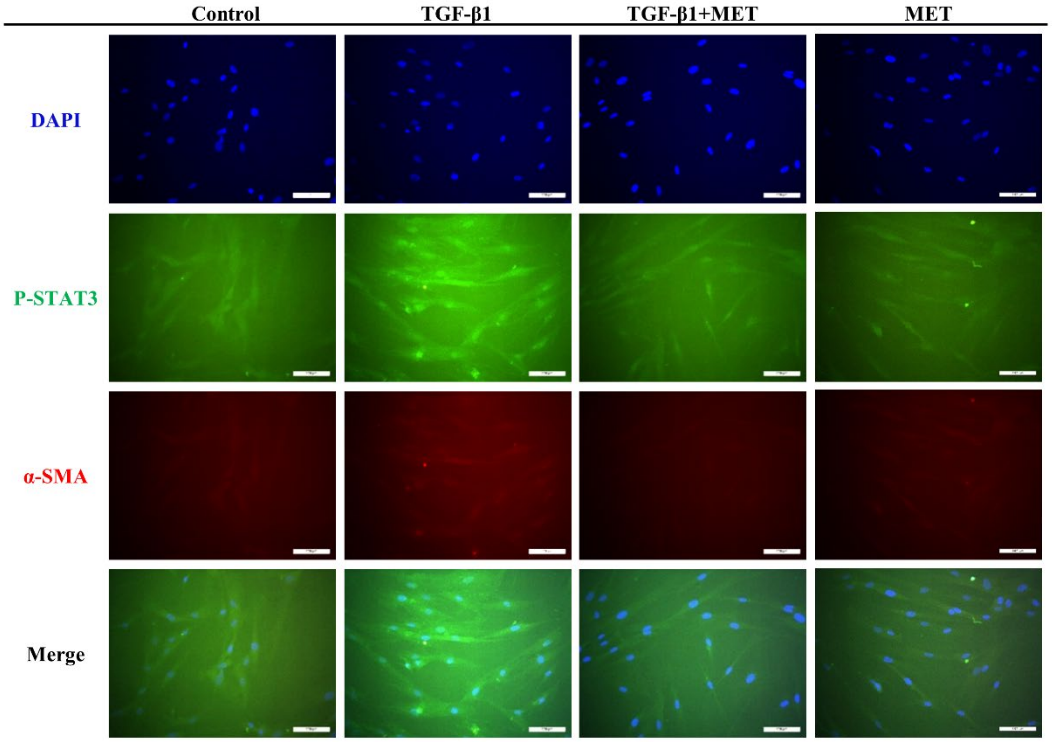

Supplementary Figure 1. HFL1 cells were pretreated with MET (10 mM) for 24 h and then incubated with TGF-β1 (10 ng/ml) for 24 h. The expression of P-STAT3 and α-SMA were measured by immunofluorescence after 24 h treatment with TGF-β1. Scale bars represent 100 μm.
